# A unified approach for the thermodynamic comparison of heat pump cycles

**DOI:** 10.1038/s44172-023-00112-0

**Published:** 2023-09-05

**Authors:** Zhibin Yu, Zahra Hajabdollahi Ouderji

**Affiliations:** James Watt School of Engineering, University of Glasgow, Glasgow, G12 8QQ UK

**Keywords:** Energy modelling, Mechanical engineering

## Abstract

The flexible heat pump cycle introduces a heat storage device into the Evans-Perkins cycle to recover, store, and reuse part of the sensible heat carried by the hot liquid refrigerant from the condenser, achieving a higher coefficient of performance than the latter. In this paper, we develop a unified approach, namely cycle superposition to allow comparison of the flexible heat pump cycle with other performance-enhancing cycle layouts including two-stage cycles with intercooling, subcooling, flash gas removal, or their combinations. We show that under ideal conditions, the flexible heat pump cycle is thermodynamically similar to two-stage heat pump cycles with full subcooling or flash gas removal, but no intercooling. From the energy recovery perspective, the two-stage cycles recover and reuse some sensible heat carried by hot liquid refrigerant simultaneously using their high-stage compressor, whereas the flexible heat pump cycle decouples the recovery and reuse of such heat in time using a heat storage. However, the irreversible heat transfer via real heat exchangers during charging and discharging processes will reduce the benefits of the flexible heat pump cycle. The effectiveness of all these performance-enhancing methods strongly depends on the characteristics of refrigerants.

## Abstract (web summary)

Yu and Ouderji compare the thermodynamic performance of the flexible heat pump cycle with other performance enhancing cycles using a unified approach. Their analysis uncovers the working mechanisms and refrigerant characteristics that improve system performance, guiding heat pump optimisation.

## Introduction

It is widely believed that heat pumps are a key technology for heat decarbonisation to reach the net zero greenhouse gas emission target by 2050, but their uptake has not been as fast as expected so far^1^. Innovations are needed to further improve their cost-effectiveness and facilitate wide deployment.

**Fig. 1:**
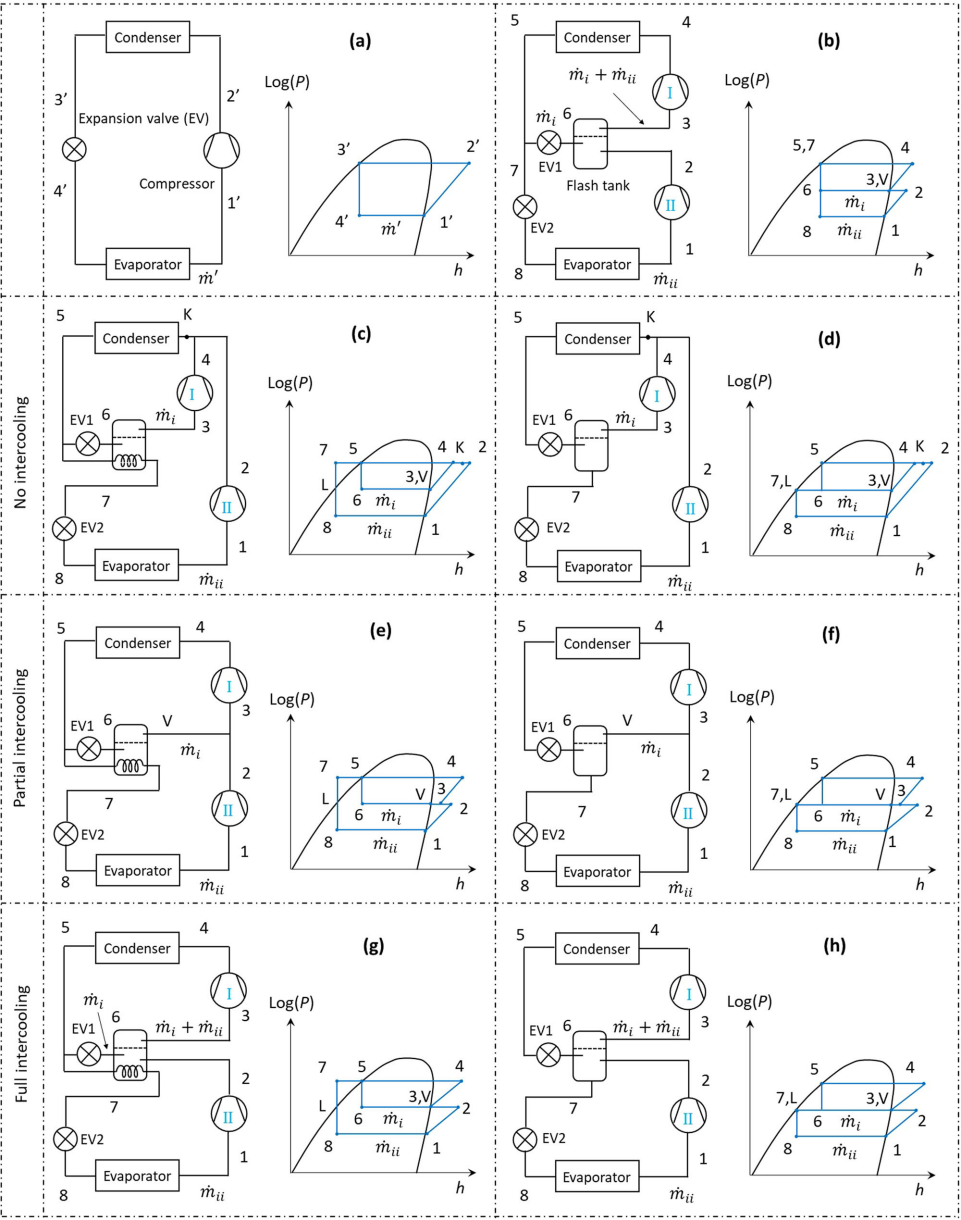
The energy performance of the standard Evans-Perkins cycle can be improved by intercooling, subcooling, flash gas removal, or the combination of them. **a** Single-stage Evans-Perkins heat pump cycle; (**b**) two-stage with intercooling (IC); (**c**) parallel compression with full subcooling (SC); (**d**) parallel compression with flash gas removal (FGR); (**e**) two-stage with full subcooling and partial intercooling (SC + PIC); (**f**) two-stage with flash gas removal and partial intercooling (FGR + PIC); (**g**) two-stage cycle with full subcooling and full intercooling (SC + FIC); (**h**) two-stage with flash gas removal and full intercooling (FGR + FIC). The state points around the cycles are represented by numbers 1–8, 1’−4’, and the letters K, L, and V.

Most heat pumps in the market are built upon a standard Evans-Perkins vapour compression cycle with or without performance enhancing mechanisms. As illustrated in Fig. 1a, the standard Evans-Perkins vapour compression cycle consists of a compressor, a condenser, an expansion device, and an evaporator. The compressor extracts low pressure refrigerant vapour from the evaporator and compresses it to the condensing pressure. The high temperature vapour exiting the compressor de-superheats and condenses in the condenser to transfer heat to a heat sink (e.g., a central heating system). The high-temperature and high-pressure liquid refrigerant leaving the condenser is then throttled to the evaporating pressure through an expansion device. The produced two-phase mixture then fully evaporates in the evaporator after absorbing heat from a heat source (e.g., outdoor air).

As the temperature lift between the heat source and sink increases, the coefficient of performance (COP) of a single-stage heat pump decreases dramatically. One key issue is that the hot liquid refrigerant leaving the condenser has a high temperature and pressure, and thus contains a substantial amount of energy, which cannot be utilised in current standard single-stage heat pumps as shown in Fig. 1a, of which the hot liquid refrigerant is normally throttled to the evaporating pressure through an expansion device. The throttling process produces a large quantity of flash gas that needs to be recompressed to the condensing pressure, wasting compressor work, and degrading the system’s COP. The larger the temperature lift, the more the flash gas, the more power wasted for recompression.

Various methods have been developed to address this issue, and they can be roughly categorised into three major groups: (1) Using a power generation device to recover energy during expansion process; (2) using a two-stage cycle to partially avoid the recompression of flash gas; (3) using a heat storage to recover some heat from the hot liquid refrigerant and then reuse it as an ancillary heat source for the heat pump’s operation, namely the flexible heat pump cycle.

Several methods for generating power from the expansion processes have been developed and demonstrated, particularly for trans-critical CO_2_ cycles.

First, expanders have been used to replace the expansion valves and recover power from the expansion process^2–4^. Many studies indicated that the turbine expanders can recover energy to reduce the irreversibility of the expansion process^5–7^, but they are mechanically complicated and expensive^8,9^. Volumetric devices such as rolling piston, screw, scroll, and piston expanders have also been considered to recover energy during expansion process^10^. Baek et al. constructed and tested a piston-cylinder type expansion device for a trans-critical CO_2_ system and reported a 10% system performance improvement^11^. However, piston cylinder expanders are not popular due to the control issue of valves^12^. Recently, Zhang et al. developed a double acting free piston expander for CO_2_ systems^13^. Li et al.^14^ suggested a rolling piston-type expander to replace the expansion valve. It was reported that their prototype achieved a maximum isentropic efficiency of 58% and improved the system’s COP by 10%. However, there was an issue with suction flow control and internal leakage in rolling piston expanders^12,15^.

Second, ejectors are another method for recovering energy from the expansion process^16,17^. They accelerate the high-pressure refrigerant from the condenser through a nozzle, converting its potential energy (i.e., pressure) into kinetic energy (i.e., velocity), and thus create a low-pressure region around the nozzle tip to suck low pressure refrigerant vapour from the evaporator, which can reduce the compressor power consumption. Ejectors have been intensively researched for trans-critical CO_2_ heat pumps and refrigerators^18^. It is reported that ejectors could improve COP of the trans-critical CO_2_ cycles by up to 16–20% in theory^19–22^.

In addition to the power recovery strategy described above, several power saving methods have been developed to partially avoid recompressing the flash gas generated during expansion. Two-stage cycles with various power saving methods have been proposed and developed for refrigeration and heat pumps in the past decades^23^, which include intercooling^24^, subcooling^25–27^, flash gas removal^28,29^, and their various combinations^30–32^. A comprehensive review of different two-stage cycle layouts for heat pumps and refrigerators can be found elsewhere^31,32^. Several typical layouts of two-stage heat pump cycles are illustrated in Fig. 1, and they are described in detail in the next section of this paper. It is predicted that a two-stage cycle with inter-stage subcooling can increase the heat pump’s COP by up to 23% in theory^31^.

Sarkar compared the potential effectiveness of these methods for improving the COP of trans-critical CO_2_ heat pumps, showing that the turbines could achieve the highest COP improvement, followed by two-stage cycles^33^. However, it should be noted that both the above-mentioned strategies introduce an extra mechanical device, either an expander or an extra compressor, to the heat pumps or refrigerators, leading to more complexity and higher costs.

Unlike these two strategies, the recently developed flexible heat pump cycle provides a new way to address the issue of high losses associated with the expansion process. It introduces a heat storage system into the standard Evans-Perkins cycle to recover, store, and reuse part of the sensible heat carried by the hot liquid refrigerant leaving the condenser, and thus can achieve a higher COP than the standard Evans-Perkins cycle. The proposed flexible heat pump cycle concept has been proved theoretically, numerically, and experimentally^34^.

According to the literature review above, there are several interesting questions to be answered: (1) How efficient is the flexible heat pump cycle compared with other performance-enhancing methods such as sub-cooling, intercooling, flash gas removal? (2) How and why does the refrigerant type affect the performance enhancement of the flexible heat pump cycle? (3) What is the underlying relationship between the flexible heat pump cycle and two-stage heat pump cycles? (4) What are the key design considerations for achieving the benefits of the flexible heat pump cycle?

Here we developed a unified approach, namely cycle superposition, to analyse various two-stage cycle layouts and the flexible heat pump cycle. Based on this, a comprehensive theoretical analysis has been conducted to compare the performance of the flexible heat pump cycle with two-stage heat pump cycles having various performance-enhancing mechanisms. The paper firstly analyses each individual performance-enhancing mechanism (i.e., intercooling, sub-cooling, and flash gas removal), and then combines them in various ways. Their effectiveness is then compared with the flexible heat pump cycle.

It is revealed that the flexible heat pump cycle and the two-stage heat pump cycles can be regarded as the superposition of two single-stage cycles. Neglecting the irreversibility of heat transfer processes, the flexible heat pump cycle is thermodynamically similar to the two-stage heat pump cycles with full subcooling or flash gas removal, but without intercooling. The more the heat that can be recovered from the low-COP component cycle to the high-COP one, the higher the COP improvement. It is also found that the effectiveness of all these performance-enhancing methods strongly depends on the characteristics of refrigerants, particularly the slopes of their saturation liquid and vapour lines.

## Results

### Two-stage heat pump cycles

Two-stage compression with intercooling is one potential way to reduce the compressor power, by bringing the compression towards an ideal isothermal compression process which requires the least power. Intercooling can be provided by an external cooling source or the evaporation of a stream of liquid refrigerant within the cycle (i.e., refrigerant flash intercooling). The former is always beneficial if free cooling sources are provided. However, refrigerant flash intercooling is more complicated as it increases the mass flow rate of the refrigerant in the high-stage compressor, and thus increases its compression power. The net effect will depend on the trade-off between these two competing effects, and thus its effect on the total compression power is very complex.

According to a literature survey, refrigerant flash intercooling was often investigated in combination with other performance-enhancing methods in two-stage cycles. It is therefore interesting to create a two-stage cycle that only has refrigerant flash intercooling to study its power saving benefit.

Figure 1b presents such a theoretical two-stage cycle with refrigerant flash intercooling only, denoted as IC thereafter. One stream (${\dot{m}}_{i}$) of the liquid refrigerant exiting the condenser is throttled through expansion valve EV1 to a flash tank at an intermediate pressure (process 5–6). The produced cold liquid refrigerant is used to de-superheat the vapour discharged by the low-stage compressor (i.e., Compressor II, process 2–3). The other stream of liquid refrigerant (${\dot{m}}_{{ii}}$) from the condenser is throttled through EV2 to evaporating pressure (Process 5 or 7 to 8). The high-stage compressor (i.e., Compressor I) compresses the combined streams of vapour from the flash tank to the condensing pressure (process 3–4).

Figure 1c illustrates a two-stage heat pump cycle with a refrigerant-based sub-cooler, denoted as SC thereafter. One stream (${\dot{m}}_{i}$) of the liquid refrigerant from the condenser is throttled to the flash tank (process 5–6), which cools the other stream of refrigerant (${\dot{m}}_{{ii}}$) to a sub-cooled state (process 5–7). The two compressors are in parallel. Compressor I compresses the vapour from the flash tank to the condensing pressure (process 3–4), while Compressor II compresses the vapour from the evaporator to condensing pressure (process 1–2). The two streams merge and mix to reach state K before entering the condenser. There is no intercooling in this cycle layout. It should be noted that the degree of subcooling (${T}_{5}-{T}_{7}$) can be arbitrary between 0 and (${T}_{5}-{T}_{6}$) inclusive. In this paper, we focus on an ideal full subcooling case, which means the refrigerant stream (${\dot{m}}_{{ii}}$) is fully cooled down to the flash tank temperature (${T}_{i}$). As a result, ${T}_{5}-{T}_{7}={T}_{5}-{T}_{6}$, or in other words ${T}_{7}={T}_{6}$.

Figure 1d illustrates a two-stage cycle with the flash tank as a flash gas removal, denoted as FGR thereafter. The liquid refrigerant from the condenser is throttled through expansion valve EV1 to the flash tank (process 5–6), where the vapour is separated and removed from liquid. The liquid is then further throttled to the evaporating pressure via EV2 (process 7-8). The two compressors are in parallel. Compressor I compresses the vapour from the flash tank to  the condensing pressure (process 3−4), while Compressor II compresses the vapour from the evaporator to the condensing pressure (process 1–2). The two refrigerant streams merge and mix to reach state K before entering the condenser. There is no intercooling in this cycle layout.

Figure 1e shows a two-stage cycle using the flash tank as a sub-cooler, where the saturated vapour from flash tank (State V) mixes with and slightly cools the vapour discharged from Compressor II (process 2–3). The high-stage compressor (Compressor I) compresses the combined vapour streams to the condensing pressure (process 3–4). This layout is denoted as SC + PIC, where PIC stands for partial intercooling.

Figure 1f illustrates a two-stage cycle using the flash tank as a flash gas removal, where the saturated vapour from flash tank (State V) mixes with and slightly cools the vapour discharged from the low-stage compressor (Compressor II), i.e., process 2–3. The high-stage compressor (Compressor I) compresses the combined vapour streams to the condensing pressure (process 3–4). Similarly, this layout is denoted as FGR + PIC.

Figure 1g presents a two-stage cycle using the flash tank as a sub-cooler and intercooler. The vapour discharged from the low-stage compressor (Compressor II) is fully cooled down to the saturated temperature (process 2–3) of the flash tank. The high-stage compressor (Compressor I) compresses the combined vapour streams to the condensing pressure (process 3–4). This layout is denoted as SC + FIC, where FIC stands for full intercooling.

Figure 1h shows a two-stage cycle using the flash tank as a flash gas removal and intercooler. The vapour discharged from the low-stage compressor (Compressor II) is fully cooled down to the saturated temperature within the flash tank (process 2–3). The high-stage compressor (Compressor I) compresses the combined vapour streams to the condensing pressure (process 3–4). Similarly, this layout is denoted as FGR + FIC.

It should be noted that the two-stage heat pump cycle layouts shown in Fig. 1 are not exhaustive. There are other possible layouts that incorporate the mentioned basic performance-enhancing methods, along with other techniques (e.g., internal heat exchangers). These alternative layouts are thermodynamically similar to the seven layouts summarised in Fig. 1, so they have been omitted from this discussion for brevity.

### Simulations

As described in “Methods” section, based on upon well-established thermodynamic modelling methods (i.e., heat and mass balance analysis under ideal conditions)^30–32^, a unified cycle superposition approach is proposed and developed to analyse various two-stage heat pump cycle layouts, along with the flexible heat pump cycle. Based on the obtained models, this section presents the numerical simulations to assess the COP improvement of various performance-enhancing methods including intercooling, sub-cooling, flash gas removal, and their combinations. The obtained results are subsequently compared with the Flexible Heat Pump cycle.

For the convenience of comparison with the Flexible Heat Pump cycle^34^, the evaporating temperature is set as 0 °C, while a list of condensing temperatures is tested from 25 to 85 °C at a step of 5 °C. The thermophysical properties are obtained using REFPROP V9.0^35^.

### Intercooling

**Fig. 2:**
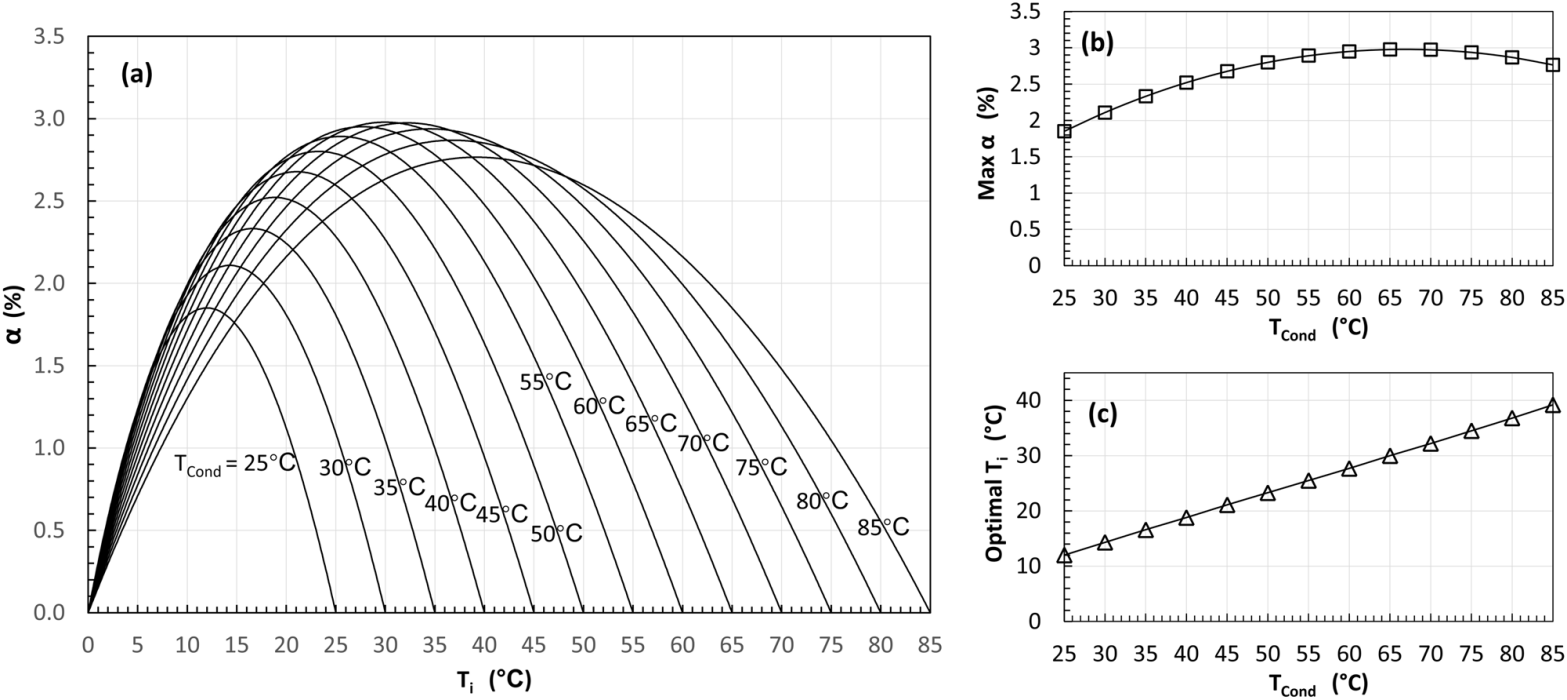
Refrigerant flash intercooling only slightly increases the coefficient of performance (COP) for ammonia. **a** The calculated COP improvement $\alpha$ varies when the intermediate temperature ${T}_{i}$ in the flash tank increases for different condensing temperatures. **b** The maximum COP improvement (Max *α*). **c** The corresponding optimal flash tank temperature (optimal ${T}_{i}$).

Figure 2a shows the calculated COP improvement $\alpha$ of the cycle layout IC (see Fig. 1b) using ammonia as a refrigerant. For each case, $\alpha$ increases and then decreases as the intermediate temperature (${T}_{i}$) in the flash tank varies from the evaporating temperature (i.e., 0 °C) to each tested condensing temperature. The calculated results resemble a bell shape like the results of the flexible heat pump cycle when the latent heat storage temperature increases^34^. However, the COP improvement is much lower than that of the flexible heat pump cycle.

The maximum COP improvement (Max *α*) and the corresponding optimal flash tank temperature (optimal ${T}_{i}$) of all cases in Fig. 2a are extracted and plotted in Fig. 2b, c, respectively. According to Fig. 2b, as the condensing temperature increases, ${{{{{\rm{Max}}}}}}\,\alpha$ increases from about 1.8% to around 3%, when the condensing temperature increases from 25 to 65 °C. It then decreases as the condensing temperature further increases from 65 to 85 °C. This may be attributed to the trade-off between the power saving due to intercooling and the power increase due to the refrigerant mass flow rate increase through the high stage compressor. As shown in Fig. 2c, the optimal ${T}_{i}$ increases near linearly as the condensing temperature increases, and it is slightly below the average temperature of the evaporation and condensing temperatures.

**Fig. 3:**
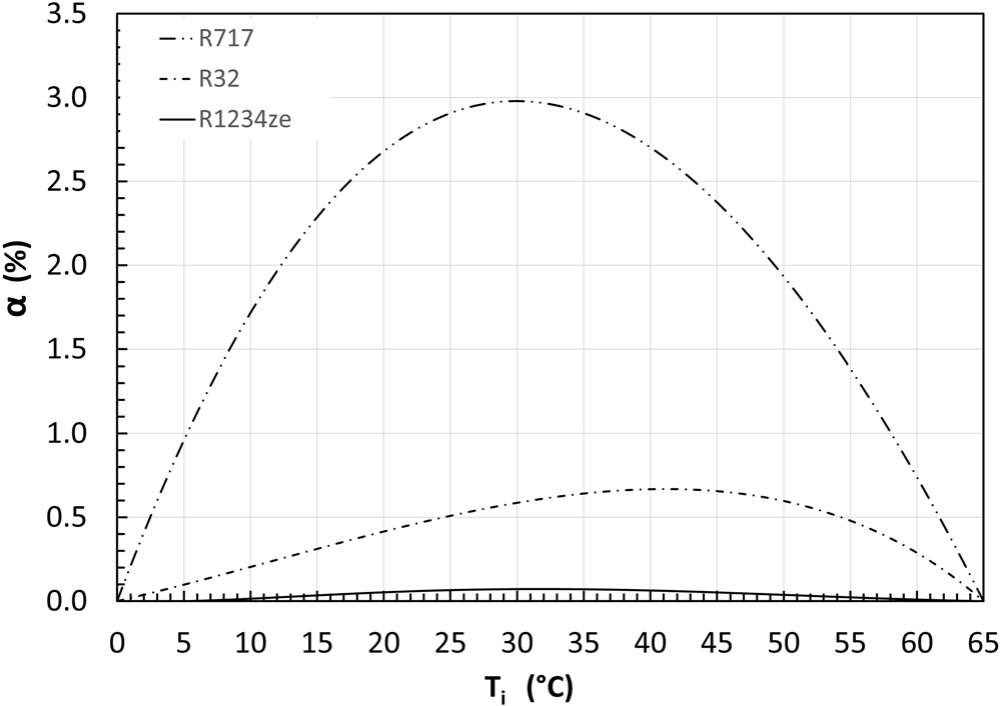
The COP improvement by refrigerant flash intercooling for three different refrigerants. The case study has an evaporating temperature at 0 °C and a condensing temperature at 65 °C. R717 and R32 are dry refrigerants, while R1234ze is a near isentropic refrigerant.

Figure 3 presents the effects of the refrigerant type on the COP improvement by refrigerant flash intercooling, for an evaporating temperature at 0 °C and a condensing temperature at 65 °C. The simulations have evaluated a list of common refrigerants, including R134a, R717, R32, R1234ze, R410a, R245fa, R227ea. Positive COP improvement is only obtained for R717, R32, R1234ze, whilst negative COP improvement is observed in the simulation results for the rest of those refrigerants. It can be inferred that the benefit of refrigerant flash intercooling strongly depends on the characteristics of refrigerants.

For dry refrigerants (see the “Methods” section for definition), there is a certain degree of superheat at the outlet of the low-stage compressor, intercooling can be applied. However, it may or may not reduce the system’s compression power, depending on the distribution of isentropes in the superheat region of the refrigerants. If the refrigerant’s isentropes diverge from each other within the superheat region, intercooling brings the high-stage compression towards the saturation line, and thus can reduce compression power, e.g., R717. However, if they are in parallel (e.g., R410a, R32, R22) to each other, intercooling does not reduce the compression power and may even increase it, reducing the systems’ COP.

For isentropic refrigerants, there is no superheat, so intercooling does not reduce the compression power. Take R1234ze as an example, the COP improvement is close to zero as shown in Fig. 3. Furthermore, for wet refrigerants, isentropic compression from a saturation vapour ends in two-phase region, which mathematically leads to a negative degree of superheat, and consequently a negative COP improvement, which is thermodynamically meaningless. Therefore, the cases with negative COP improvement due to intercooling for wet refrigerant are omitted from Fig. 3.

However, in real systems, several degrees of superheat are always necessary to avoid wet compression and protect the compressors. Intercooling can reduce discharging temperature from the high-stage compressor, which is desirable for avoiding overheating the compressors in real systems.

The impact of refrigerant type on the benefits of refrigerant flash intercooling has rarely been studied in the open literature. Jiang et al. studied several two-stage cycles which combined intercooling with subcooling or flash gas removal^31^. They reported that intercooling improves the COP for refrigerants with small specific heat but reduces the heat pump’s COP for other refrigerants with large specific heat. They commented that the larger the specific heat, the less temperature increase during compression. They concluded that reducing the discharging temperature through intercooling could not significantly reduce the specific compressor power but would increase the mass flow rate of the refrigerant through the high-stage compressor, and thus increase the compressors’ power consumption. This analysis correctly pointed out the competing effect between the potential compression power saving due to intercooling and the consequent compression power increase at the higher-stage compressor.

However, their study seems to have inadvertently overlooked an important aspect that there is no/little superheat at the outlet of the low stage compressor for isentropic and wet refrigerants (see more details in the “Methods” section). Since there is no superheat at the outlet of the low-stage compressor for isentropic and wet refrigerants, intercooling could potentially lead to a negative COP improvement, in other words a reduction of COP.

Nevertheless, as shown by the results above, the COP improvement can be achieved by refrigerant flash intercooling is marginal, compared with flexible heat pump cycle^34^.

### Subcooling

**Fig. 4:**
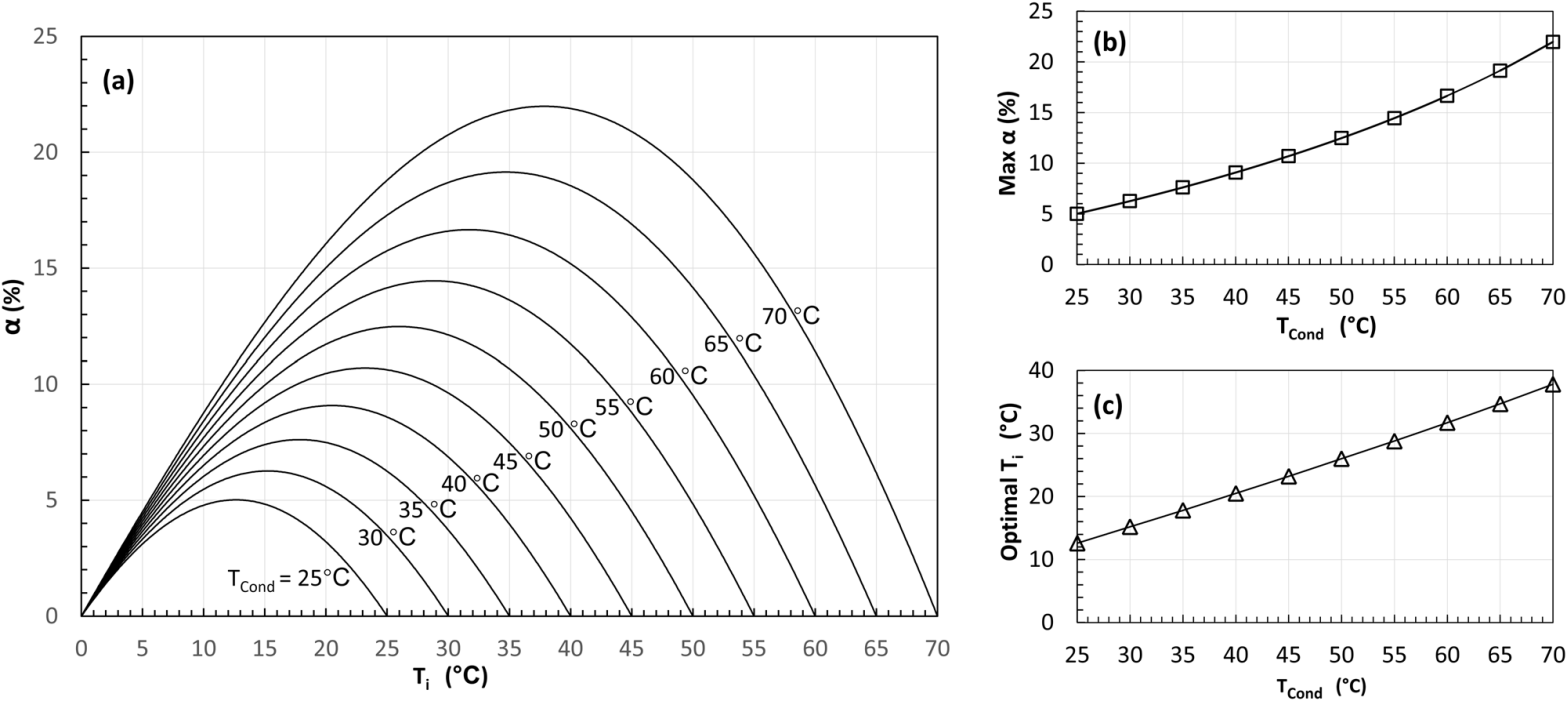
Simulation results of the two-stage cycle with subcooling (SC). In this case study, considering R134a as the refrigerant, the condensing and evaporating temperatures are 65 °C and 0 °C, respectively. The results are similar to the Flexible Heat Pump cycle^34^. **a** The calculated COP improvement $\alpha$ varies when the intermediate temperature ${T}_{i}$ in the flash tank increases for different condensing temperatures. **b** The maximum COP improvement (Max *α*) increases as the condensing temperature increases. **c** The corresponding optimal flash tank temperature (optimal ${T}_{i}$) increases as the condensing temperature increases.

For the two-stage cycle with full subcooling (SC) as shown in Fig. 1c, the calculated COP improvement $\alpha$ is shown in Fig. 4a. As defined above, for a full subcooling case, the refrigerant stream (${\dot{m}}_{{ii}}$) is fully cooled down to the flash tank temperature (${T}_{i}$). It can be found that the COP improvement $\alpha$ increases and then decreases as ${T}_{i}$ increases from the evaporating temperature to the condensing temperature.

The maximum $\alpha$ and the corresponding optimal ${T}_{i}$ of all cases are extracted and plotted against the condensing temperatures as shown in Fig. 4b, c, respectively. As can be seen from Fig. 4b, the maximum $\alpha$ increases from 5% to about 22% as the condensing temperature increases from 25 to 70 °C. As shown in Fig. 4c, the optimal ${T}_{i}$ is always close to the average of evaporating and condensing temperatures and increases linearly as the condensing temperature increases. The results shown in Fig. 4 are identical to the results of the Flexible Heat Pump cycle in the ref. ^34^.

**Fig. 5:**
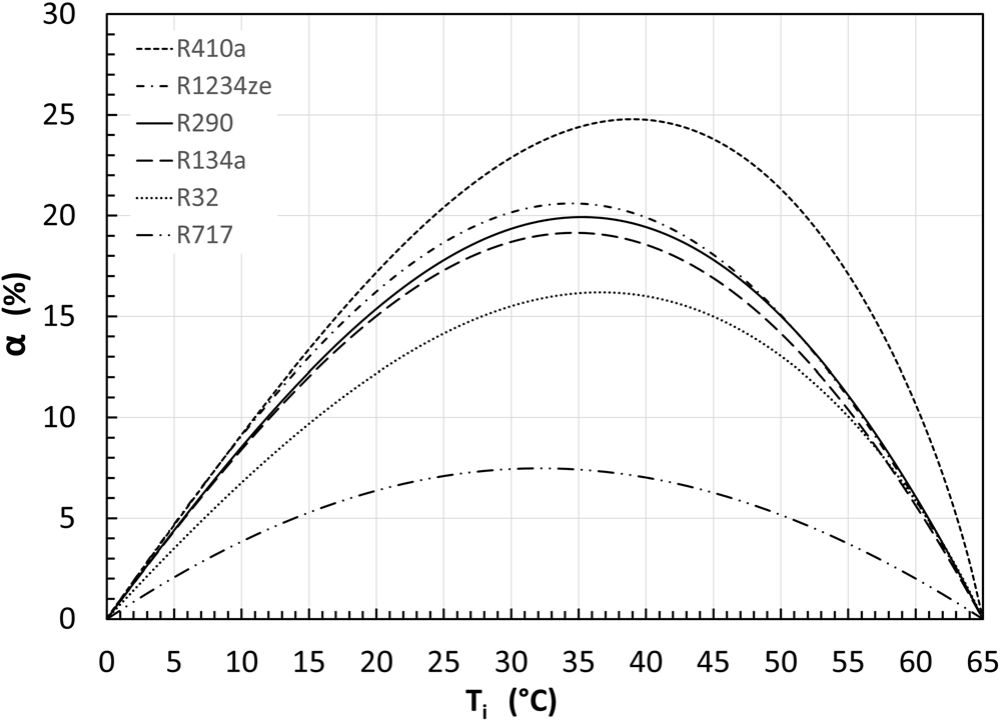
The impact of refrigerant types. The COP improvement of the two-stage cycle with subcooling (SC) varies for different refrigerants. In this case study, the condensing and evaporating temperatures are 65 °C and 0 °C, respectively. Six common refrigerants were tested. The results are identical to the flexible heat pump as shown in ref. ^34^.

Similarly, the analysis has also assessed a list of other common refrigerants for comparison through a case study, with condensing temperature at 65 °C and evaporating temperature at 0 °C. The calculated results are shown in Fig. 5. It can be found that refrigerant-based subcooling can improve heat pumps’ COP for all tested refrigerants. Interestingly, the best COP improvement $\alpha$ can be achieved using R410a and the least $\alpha$ can be achieved using R717. The results shown in Fig. 5 are identical to the results of Flexible Heat Pump cycle in the ref. ^34^. The impact of the refrigerant type on the COP improvement provided by subcooling will be analysed and discussed explicitly later in this paper when the heat production contribution ratio is discussed.

### Flash gas removal

As derived in the “Methods” section, the mathematical model of the two-stage cycle layout FGR is the same as the layout SC with full subcooling (i.e., ${T}_{6}={T}_{7}$). As shown in Fig. 1c, d, once the temperatures in the condenser, flash tank, and evaporator are given, both systems will have the same ${h}_{1}$,${h}_{3}$, ${h}_{5}$, and ${h}_{L}$. Consequently, they have the same, ${h}_{6}$, ${h}_{7}$ and ${h}_{8}$. As a result, for a given heating capacity, both cycles should have the same ${\dot{m}}_{i}$ and ${\dot{m}}_{{ii}}$. Therefore, the FGR cycle as shown in Fig. 1d is thermodynamically similar to the SC cycle with full subcooling as shown in Fig. 1c. The calculated results of the FGR cycle are similar to the SC cycle, and hence are omitted here.

### Comparison with Flexible Heat Pump cycle

The analysis has then been conducted for all other two-stage cycle layouts as shown in Fig. 1e–h for all the six selected refrigerants to compare their COP improvement against the single-stage cycle as shown in Fig. 1a under the same operational conditions. The obtained results are all very similar to Figs. 4, 5.

**Fig. 6:**
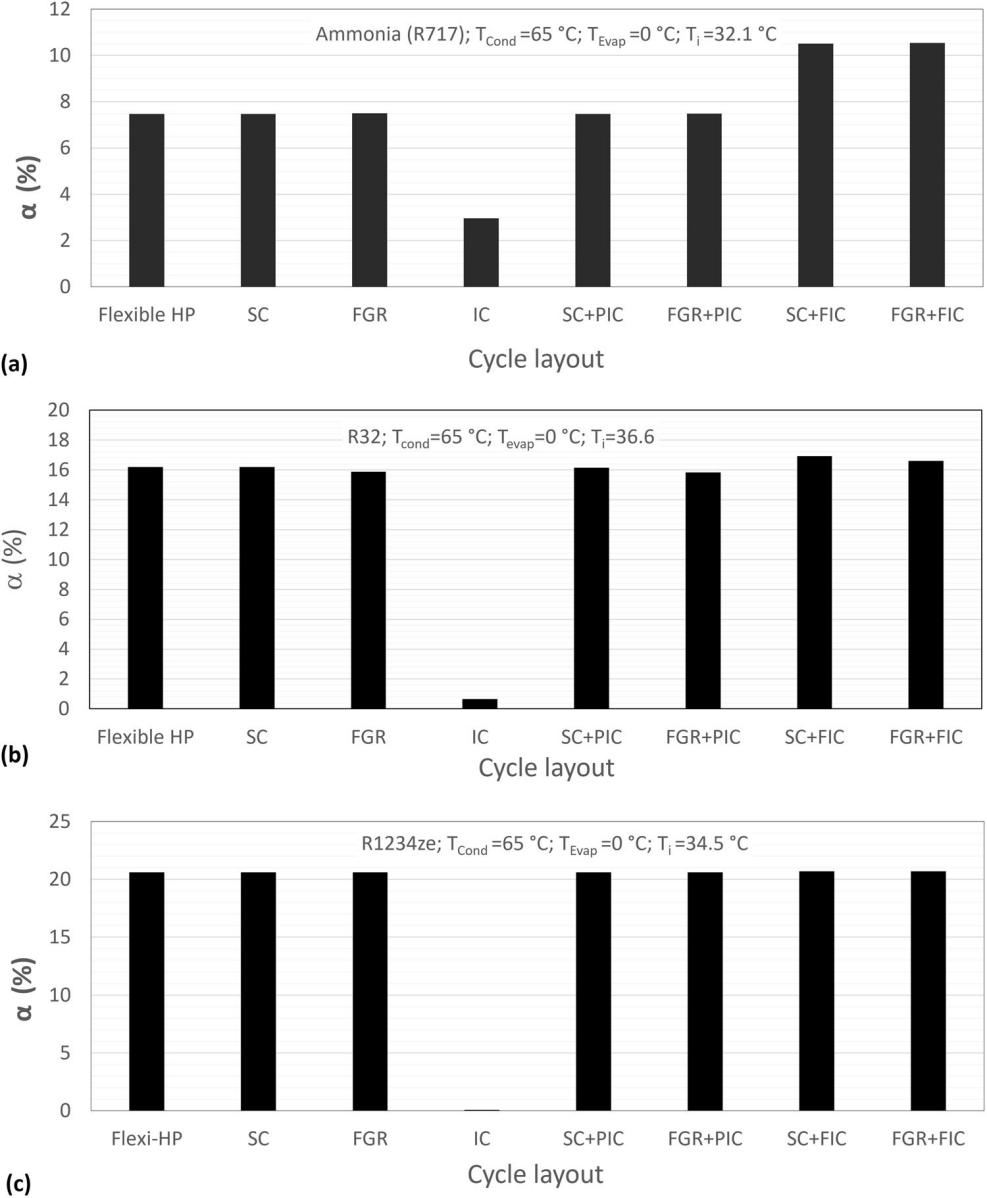
The comparison of COP improvement of flexible heat pump cycle and various two stage cycle layouts. In all cases, the condensing temperature and evaporating temperature are kept at 65 °C and 0 °C, respectively, while the intermediate temperature is selected as the optimal temperature for each refrigerant according to Fig. 3. Three typical refrigerants are tested: (**a**) R717; (**b**) R32; (**c**) R1234ze.

For the convenience of comparison, we selected one exemplar case with a condensing temperature at 65 °C and an evaporating temperature at 0 °C for all the layouts as shown in Fig. 1 and for all the six selected refrigerants used in Fig. 5. The optimal point of each case is then extracted for comparison. The results of three typical refrigerants R717, R32, and R1234ze, for which the benefits of refrigerant flash intercooling are not negative, are shown in Fig. 6.

Figure 6a shows the COP improvement $\alpha$ of all performance-enhancing methods for refrigerant R717 (i.e., ammonia). It can be seen that $\alpha$ is the same for subcooling (SC), flash gas removal (FGR) and Flexible Heat Pump (Flexi-HP) cycles, being about 7.5%. For intercooling, $\alpha$ is about 3%. The two cycle layouts with partial intercooling, SC + PIC and FGR + PIC, have almost the same $\alpha$ of around 7.5%, showing the intercooling provided by the saturated vapour from the flash tank is negligible. The two cycle layouts with full flash intercooling, SC + FIC and FGR + FIC, have the same $\alpha$ of around 10.5%, which is the sum of the COP improvement of sub-cooling (or flash gas removal) and the intercooling. The results proved the theoretical prediction as described in the “Methods” section, i.e., the combined effect of two performance-enhancing methods, subcooling and intercooling (or flash gas removal and intercooling), are their linear superposition.

Figure 6b presents the results for refrigerant R32. Flexi-HP and SC have the same COP improvement $\alpha =16.2 \%$. For FGR layout, $\alpha =15.9 \%$, which is very close to that for SC cycle as the theoretical analysis predicted. The tiny but noticeable difference is unexpected according to the models, and it might be attributed to the inaccuracy of thermophysical properties using REFPROP^35^. For cycle IC, $\alpha =0.65 \%$, implying that the refrigerant flash intercooling has little benefit. The two partially intercooled cycles (SC + PIC and FGR + PIC) have the same COP improvement as SC and FGR, respectively. For SC + FIC, $\alpha =16.9 \%$ which is roughly the sum of COP improvements for SC (16.2%) and IC (0.65%) cycles. Similarly, for FGR + FIC, $\alpha =16.6 \%$ which is roughly the sum of the COP improvement for FGR (15.9%) and IC (0.65%) cycles.

Figure 6c presents the results for R1234ze. For the IC, $\alpha \approx 0$, implying that the refrigerant flash intercooling does not change the COP against the standard single-stage system. All the other cycle layouts have almost the same COP improvement $\alpha$ of 20.6%.

Figure 6 also demonstrates that the refrigerant type has a strong impact on the COP improvement of all seven performance enhancing methods. The trend agrees with the observations of Figs. 5, 3. The underlying mechanism will be explicitly discussed in the following section.

### Ratio of heat production contributed by two component cycles ${{{{\boldsymbol{\epsilon}}}}}$

**Fig. 7:**
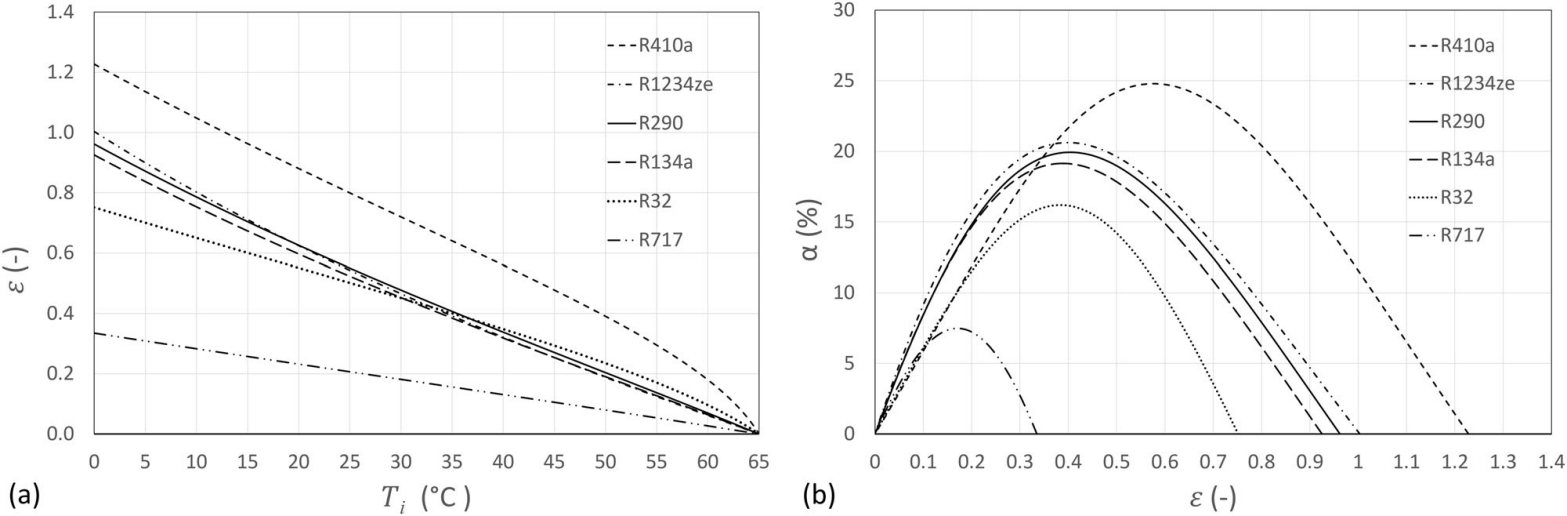
The SC cycle layout with an evaporating temperature of 0 °C and a condensing temperature of 65 °C. **a** The calculated ${{{{{\rm{\varepsilon }}}}}}$ as the flash tank temperature ${{{{{{\rm{T}}}}}}}_{{{{{{\rm{i}}}}}}}$ increases; (**b**) the calculated COP improvement ${{{{{\rm{\alpha }}}}}}$ against the calculated heat contribution ratio ${{{{{\rm{\varepsilon }}}}}}$. There exists an optimal ${{{{{\rm{\varepsilon }}}}}}$ for each case.

According to the theoretical analysis in the “Methods” section, the ratio of heat production contributed by two component cycles, $\varepsilon$, is a key parameter that influences the COP improvement. In this section, a case study is presented to show how $\varepsilon$ affects the COP improvement $\alpha$. The SC cycle layout as shown in Fig. 1c is studied with the list of refrigerants as assessed in Fig. 5. The condensing and evaporating temperatures are set as 65 °C and 0 °C, respectively. The flash tank temperature ${T}_{i}$ increases from the evaporating temperature to the condensing temperature at a step of 5 °C. For each ${T}_{i}$, The values of $\alpha$ and $\varepsilon$ are calculated and presented in Fig. 7 below.

As shown in Fig. 7a, the calculated heat contribution ratio $\varepsilon$ decreases as the flash tank temperature increases from the evaporating temperature of 0 °C to the condensing temperature of 65 °C for all the tested refrigerants. Take R134a as an example, as ${T}_{i}$ increases from 0 °C to 65 °C, $\varepsilon$ decreases from 0.93 to 0. This is because, as ${T}_{i}$ increases, less refrigerant (${\dot{m}}_{i}$) will be required to evaporate in the flash tank to cool the other stream (${\dot{m}}_{{ii}}$) down to ${T}_{i}$. Therefore, when ${T}_{i}\to {T}_{{cond}}$, ${\dot{m}}_{i}\to 0$, and thus $\varepsilon \to 0$.

The relationship between COP improvement $\alpha$ and $\varepsilon$ is plotted in Fig. 7b. Take R134a as an example, as $\varepsilon$ increases, $\alpha$ increases from 0 to the maximum 19.14%, and then decreases to 0. The shape of the curve resembles a bell shape like those shown in Fig. 5. There exists an optimal $\varepsilon$ at around 0.388, and the corresponding optimal temperature is 34.7 °C. This can be attributed to the trade-off between the quantity and quality of the heat recovered by the refrigerant stream ${\dot{m}}_{i}$ from ${\dot{m}}_{{ii}}$. Reducing ${T}_{i}$ can increase the heat recovered from the refrigerant stream ${\dot{m}}_{{ii}}$, but decrease the temperature of the recovered heat, and vice versa.

To further investigate how the refrigerant type affects the COP improvement $\alpha$. The optimum points in Figs. 7b, 5 have been extracted and the obtained results are summarised in Table 1. As discussed above, similar results were obtained for the FGR cycle layout. Following the same procedures, the optimum points for FGR were also calculated and listed in the table. In addition, for comparison, the optimum points of the flexible heat pump cycle^34^ were also extracted and listed in the table. There are several interesting observations from the results presented in the table.

**Table 1:** Comparison between flexible heat pump cycle and two stage heat pump cycles with full subcooling or flash gas removal.

Refrigerant	R410a	R1234ze	R290	R134a	R32	R717
${T}_{{HS}}$ or ${T}_{i}$ (°C)	39.0	34.5	35.3	34.7	36.6	32.1
Flexible Heat Pump Cycle						
$\alpha$ (%)	24.740	20.612	19.933	19.143	16.199	7.476
${\varepsilon }_{{flexi}}$ (-)	0.464	0.404	0.388	0.376	0.309	0.150
Parallel compression with full subcooling (SC)						
$\alpha$(%)	24.790	20.613	19.933	19.143	16.199	7.476
$\varepsilon$ (-)	0.464	0.404	0.388	0.376	0.309	0.150
Parallel compression with flash gas removal (FGR)						
$\alpha$(%)	24.112	20.624	19.875	19.116	15.882	7.498
$\varepsilon$(-)	0.450	0.404	0.387	0.375	0.303	0.150

Firstly, for all the tested refrigerants, the results of the flexible heat pump cycle are almost identical to those for the two-stage cycle with full subcooling (i.e., SC), which are also very close to the results of the two-stage with flash gas removal (i.e., FGR) cycle. It can be inferred that there is a fundamental similarity among these cycles.

Secondly, the ranking of the COP improvement for the tested refrigerants perfectly agrees with the ranking of the heat production contribution ratio $\varepsilon$ (or ${\varepsilon }_{{flexi}}$ in the case of flexible heat pump cycle). It can be inferred that higher COP improvement can be achieved by the refrigerants with a higher heat production contribution ratio $\varepsilon$.

According to Eqs. (16) and (33), for qualitative analysis, we can approximate $\varepsilon \cong \beta$ for all refrigerants, hence it can be inferred that refrigerants with a higher mass flow ratio $\beta$ can potentially achieve higher COP improvements using two-stage cycles (with SC or FGR) or the flexible heat pump cycle. According to Eq. (22), $\beta$ depends on the quality of refrigerant at the exit of the high stage expansion valve EV1 (i.e., ${x}_{6}$). The higher ${x}_{6}$ the higher $\beta$, and the higher the COP improvement. Hence, the flexible heat pump or the two-stage heat pump cycles with SC or FGR can achieve higher COP improvements for wet and isentropic refrigerants. The smaller the slope of their saturated vapour line, the higher the value of ${x}_{6}$, and the higher the COP improvement.

## Discussion

### Cycle similarity

As revealed by the results above, under ideal conditions, the flexible heat pump cycle is thermodynamically similar to the two-stage heat pump cycle with full subcooling or flash gas removal, but without intercooling. Essentially, such two-stage cycles recover and reuse the sensible heat carried by the hot liquid refrigerant from the condenser simultaneously through the high-stage compressor, while the flexible heat pump cycle decouples the recovery and reuse in time through the heat storage. The flexible heat pump cycle requires only one compressor, and thus simpler and potentially cheaper to build. The intermediate temperature (or pressure) of the two-stage cycle affects the cycle’s COP in the same way as the heat storage temperature impacts the flexible heat pump cycle, which further confirms the similarity between them.

Furthermore, from the energy recovery perspective, subcooling, flash gas removal, and flexible heat pump cycle all recover some sensible heat from the hot liquid refrigerant exiting the condenser to avoid it being degraded and then upgraded again. From the compressor work perspective, they all partially avoid the recompression of flash gases, and thus can save compressor power. These two observations are the two sides of the same coin.

### Implications for real systems

It should be highlighted that idealised thermodynamic models used in this paper neglected heat, pressures, friction losses. The calculated results indicate the maximum possible COP in theory. There are limitations when using these results.

Although the idealised models predict that subcooling, flash gas removal, and flexible heat pump cycle could achieve similar COP improvements. In the flash gas removal cycle (FGR), the heat transfers from the low COP cycle (${\dot{m}}_{{ii}}$) to the high COP cycle (${\dot{m}}_{i}$) through the evaporation process which is a phase change process involving no heat exchanger. In the subcooling cycle (SC), heat transfers from the low COP cycle (${\dot{m}}_{{ii}}$) to the high COP cycle (${\dot{m}}_{i}$) through a liquid-to-liquid heat exchanger (e.g., the flash tank with a heat exchanger as shown in Fig. 1c). In the flexible heat pump cycle, heat is firstly transferred from the liquid refrigerant to the heat storage during the charging mode and is then transferred back to liquid refrigerant during the discharging mode. Exergy destruction due to irreversibility happens in heat exchangers in real systems, which reduces the system’s performance.

It is therefore interesting to investigate how irreversible heat transfer via a real heat exchanger affects the COP improvement α of the flexible heat pump cycle. In a real flexible heat pump system, the hot liquid refrigerant from the condenser leaves the latent heat storage with a temperature higher than the melting temperature of the phase change material after it transfers heat to the phase change material during the charging mode, and the temperature difference can be denoted as ${\Delta T}_{{charge}}$. Similarly, refrigerant leaves the heat storage at a temperature lower than the melting temperature of the phase change material during the discharging mode, and the temperature difference can be denoted as ${\Delta T}_{{discharge}}$. For phase change materials, normally ${\Delta T}_{{discharge}} > {\Delta T}_{{charge}}$ due to the poorer thermal conductivity during the solidification.

**Fig. 8:**
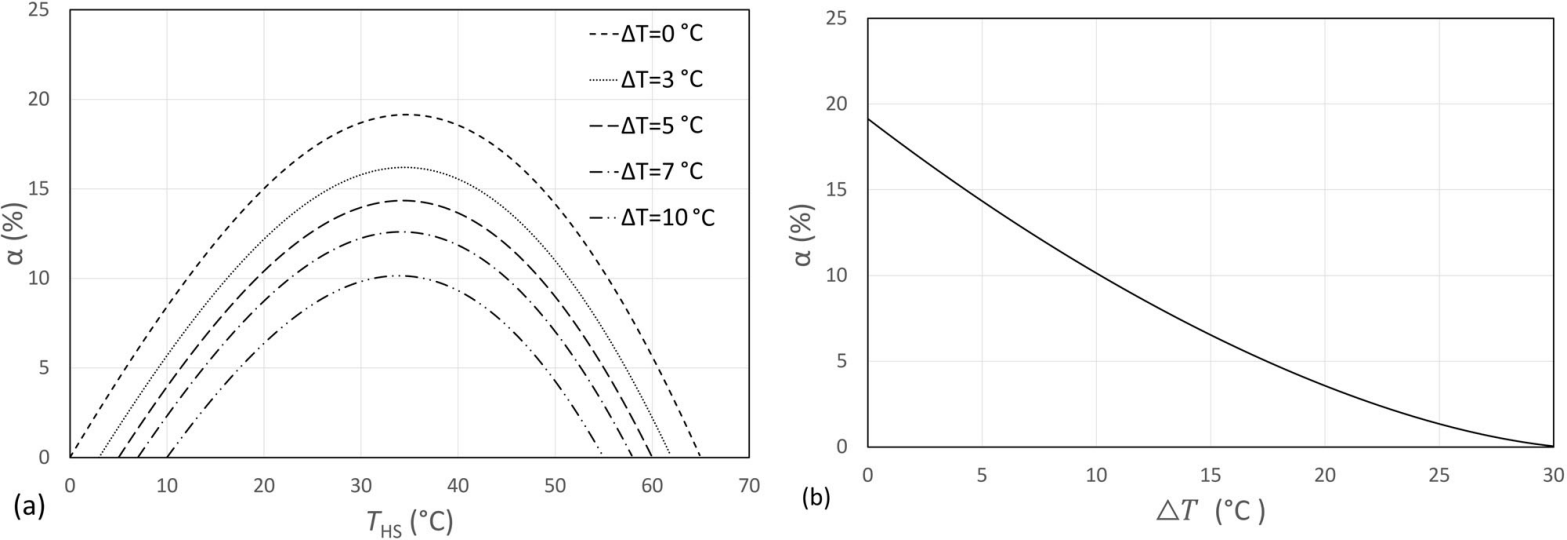
The impact of irreversible heat transfer losses within the heat storage during charging and discharging. **a** The COP improvement varies when the latent heat storage temperature increases for different temperature drops $\Delta T$. **b** The COP improvement decreases as the temperature drop within the heat storage $\Delta T$ increases.

For the convenience of analysis, we could simplify ${\Delta T}_{{discharge}}={\Delta T}_{{charge}}=\Delta T$ and conduct a case study with refrigerant R134a. The condensing and evaporating temperatures are 65 and 0 °C, respectively. Figure 8 shows how the COP improvement changes when $\Delta T$ varies. The results of the optimal points shown in Fig. 8a can be extracted and plotted in Fig. 8b. It clearly shows that, as the temperature difference $\Delta T$ increases, the COP improvement of the flexible heat pump decreases, and reaches 0 when the $\Delta T$ increases to 30 °C, at which the liquid refrigerant leaves the thermal storage at the same temperature as the condenser so no heat can be recovered, and thus no COP improvement.

It can therefore be inferred that, in real systems, the flexible heat pump cycle will be less efficient than two-stage heat pump cycles with subcooling or flash gas removal (i.e., SC or FGR cycle layouts). Hence, to achieve the potential power saving benefits of the flexible heat pump cycle in real systems, it is crucial to optimise the heat exchangers to reduce the exergy destruction during the charging and discharging of the heat storage.

### The relationship between ${{{{{\boldsymbol{\varepsilon }}}}}}$ and ${{{{{\boldsymbol{\beta }}}}}}$ for different refrigerant types

**Fig. 9:**
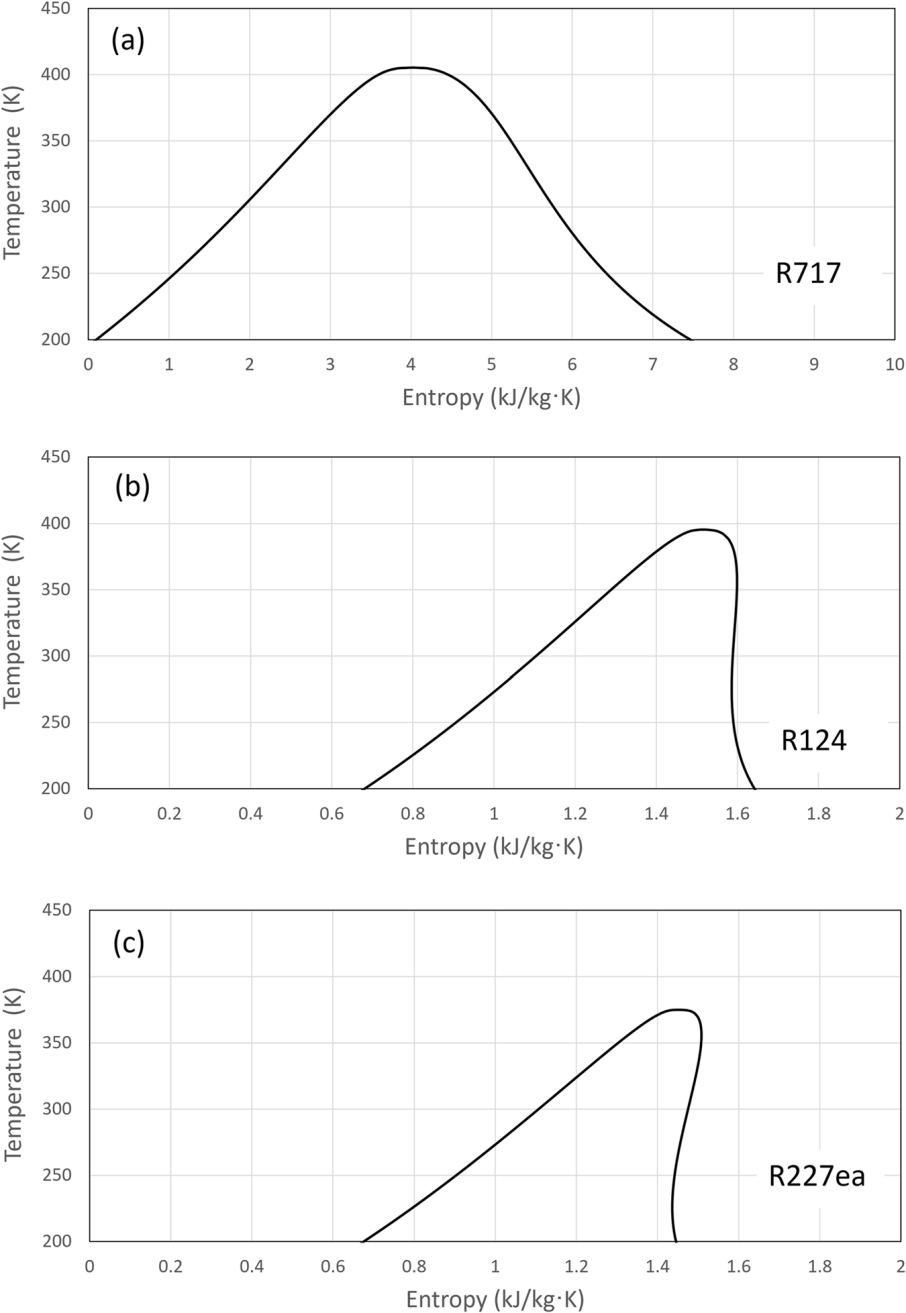
Refrigerants with different slopes of the saturation vapour line. **a** Dry refrigerant, e.g., R717; (**b**) near isentropic refrigerant, e.g., R124; (**c**) wet refrigerant, e.g., R227ea.

For the three exemplar refrigerants as shown in Fig. 9, the values of $\varepsilon$ and $\beta$ for different cycle layouts at their optimal points are calculated and summarised in Table 2. For all three refrigerants, ${\varepsilon }_{{flexi}}={\beta }_{{flexi}}$ for the flexible heat pump cycle, which agrees with the prediction by Eq. (39).

**Table 2:** The calculated values of ${{{{{\rm{\beta }}}}}}$ and ${{{{{\rm{\varepsilon }}}}}}$ for different cycle layouts using dry, near isentropic, and wet refrigerants.

Cycle	Flexi-HP	SC	FGR	SC + PIC	FGR + PIC	SC + FIC	FGR + FIC	IC
R717, *T*_*HS*_ or *T*_*i*_ = 32.1 *°*C								
β (or ${\beta }_{{flexi}}$)	0.1502	0.1709	0.1715	0.1709	0.1715	0.2988	0.2994	0.1279
ε (or ${\varepsilon }_{{flexi}}$)	0.1502	0.1502	0.1506	0.1538	0.1543	0.2684	0.2689	0.1149
R124, *T*_*HS*_ or *T*_*i*_ = 34.0 *°*C								
β (or ${\beta }_{{flexi}}$)	0.3635	0.3592	0.3598	0.3592	0.3598	0.3470	0.3477	−0.0129
$\varepsilon$ (or ${\varepsilon }_{{flexi}}$)	0.3635	0.3635	0.3642	0.3631	0.3638	0.3508	0.3515	−0.0122
R227ea, *T*_*HS*_ or *T*_*i*_ = 35.3 *°*C								
β (or ${\beta }_{{flexi}}$)	0.6379	0.5629	0.5624	0.5629	0.5624	0.4414	0.4410	−0.1215
$\varepsilon$ (or ${\varepsilon }_{{flexi}}$)	0.6379	0.6379	0.6373	0.6310	0.6305	0.4944	0.4939	−0.1361

For dry refrigerant R717, $\varepsilon \, < \, \beta$ for all two-stage cycles, which agrees with the prediction by Eq. (30). For near isentropic refrigerant R124, $\varepsilon \cong \beta$ for all two-stage cycles, which agrees with the prediction by Eq. (28). For wet refrigerant R227ea, $\varepsilon \, > \, \beta$ for all two-stage cycles, which agrees with the prediction by Eq. (32).

It is also interesting to observe that, for the two-stage cycle layout IC, negative values of $\beta$ are observed using R227ea which is a wet refrigerant. The values of $\beta$ are nearly zero for R124 which is a near isentropic refrigerant. They are positive for R717 which is a dry refrigerant. These observations perfectly agree with the prediction according to Eq. (22).

### Cycle superposition

Two-stage heat pump cycles and the flexible heat pump cycle can be regarded as the superposition of two single-stage cycles. For two-stage heat pump cycles, the two cycles are coupled through a flash tank (or other similar heat exchangers). In the flexible heat pump cycle, they are coupled via a heat storage tank.

Furthermore, the two-stage heat pump cycles that combine subcooling (or flash gas removal) with intercooling are normally dominated by the subcooling (or flash gas removal). The combined COP improvement is almost the linear supposition of both performance enhancing methods. As shown in Fig. 6, the total COP improvement is the sum of two individual COP improvements. Moreover, as shown in Table 2, $\beta$ for SC + FIC (or FGR + FIC) is the sum of $\beta$ for SC (or FGR) and IC. This agrees with the prediction by Eq. (22), which further confirms the nature of superposition between these performance enhancing methods.

### Limitation

The idealised models only consider the thermodynamic processes of the refrigerants. The cycles’ interactions with external heat transfer fluids such as air or water are not considered. The power consumption associated with external heat transfer fluids may differ for two-stage, single-stage and flexible heat pump cycles although it is normally insignificant compared with the compressor power. More advanced models and experimental tests are required to understand their impacts on the benefits of all those performance-enhancing methods.

## Conclusions

In this paper, a cycle superposition method is proposed and developed to analyse the flexible heat pump cycle and two-stage heat pump cycles with different performance-enhancing methods such as intercooling, subcooling, flash gas removal, and their combinations. The two-stage cycles and flexible heat pump cycle can be regarded as the linear superposition of two component single-stage cycles. The key scientific findings are summarised as follows:

Firstly, it is revealed that, under ideal conditions, the flexible heat pump cycle is thermodynamically similar to the two-stage cycle with full subcooling or flash gas removal, but without intercooling.

Secondly, two stage heat pump cycles recover and reuse some of the sensible heat carried by the hot liquid refrigerant simultaneously using a high-stage compressor, while the flexible heat pump cycle decouples the recovery and reuse in time using a heat storage system. Essentially, both the flexible cycle and these two-stage cycles can all partially avoid the recompression of flash gases generated during the throttling processes, and thus can save compression power.

Thirdly, it should be noted that these cycles require heat transfer from the low COP cycle/mode to the high COP cycle/mode. The more heat transfer steps are required, the less COP improvement can be achieved. Hence, the flexible heat pump cycle is expected to be less efficient than two-stage heat pump cycles with full subcooling or flash gas removal in real systems. It is thus important to optimise the heat exchangers to minimise their exergy destruction during charging and discharging the flexible heat pump cycle’s heat storage.

Finally, the refrigerant type has a strong impact on the effectiveness of all these performance-enhancing methods. The flexible heat pump cycle and two-stage cycles with SC and FGR layouts can achieve a better COP improvement for wet or isentropic refrigerants which generate more flash gas during the throttling processes than dry refrigerants.

## Methods

In this section, a unified cycle superposition modelling method is proposed and developed to analyse the performance of various two-stage heat pump cycles and the flexible heat pump cycle, allowing us to compare the flexible heat pump cycle with several existing performance enhancement methods such as intercooling, subcooling, flash gas removal, and their various combinations.

The assumptions include:

1. Heat transfer takes place under isothermal condition in all heat exchangers.
2. Compressors have 100% isentropic efficiency.
3. No heat and pressure losses through pipes and heat exchangers.
4. The heating capacity is the same for all single- and two-stage cycles.

### Single-stage cycle

For the single-stage Evans-Perkins cycle as shown in Fig. 1a, the heating capacity (${\dot{Q}}_{c,{ss}}$) of the condenser can be expressed as

1 $${\dot{Q}}_{c,{ss}}=\dot{m}^{\prime} ({h}_{2{\prime} }-{h}_{3{\prime} }),$$

where $\dot{m}$ represents mass flow rate of refrigerant, $h$ represents enthalpy, subscript ss represents single-stage, subscript ${\prime}$ indicates single stage.

The heat transfer rate of the evaporator can be expressed as

2 $${\dot{Q}}_{e,{ss}}=\dot{m}^{\prime} ({h}_{1{\prime} }-{h}_{4{\prime} }).$$

The power consumed by the compressor can be calculated as

3 $${\dot{W}}_{{comp},{ss}}=\dot{m}^{\prime} ({h}_{2^{\prime} }-{h}_{1^{\prime} }).$$

As a result, coefficient of performance of the single stage heat pump, ${{COP}}_{{ss}}$, can be expressed as

4 $${{COP}}_{{ss}}=\frac{{\dot{Q}}_{c,{ss}}}{{\dot{W}}_{{comp},{ss}}}.$$

### Two-stage cycles

As shown in Fig. 1b–h, there are seven different layouts of two-stage heat pump cycles with different performance-enhancement methods, including intercooling, subcooling, flash gas removal, and their various combinations.

So far, to analyse two-stage (or multi-stage) heat pump cycles^30–32^, individual components are often isolated from the system and their energy and mass balance are then analysed. Through the interface conditions between adjacent components, the whole cycle can then be finally solved.

In this paper, a new approach, namely cycle superposition, is proposed to analyse two-stage heat pump cycles. Each of such two-stage heat pump cycles is regarded as the superposition of two component cycles, of which the first cycle extracts heat from the heat source via the evaporator and rejects heat to the sink via the condenser, while the second cycle recovers heat from hot liquid refrigerant exiting the condenser of the first cycle through a flash tank (or an internal heat exchanger) and also rejects heat to the sink via the same condenser. As a result, unlike traditional approach analysing each component, each stream of refrigerant is analysed using this approach.

**Fig. 10:**
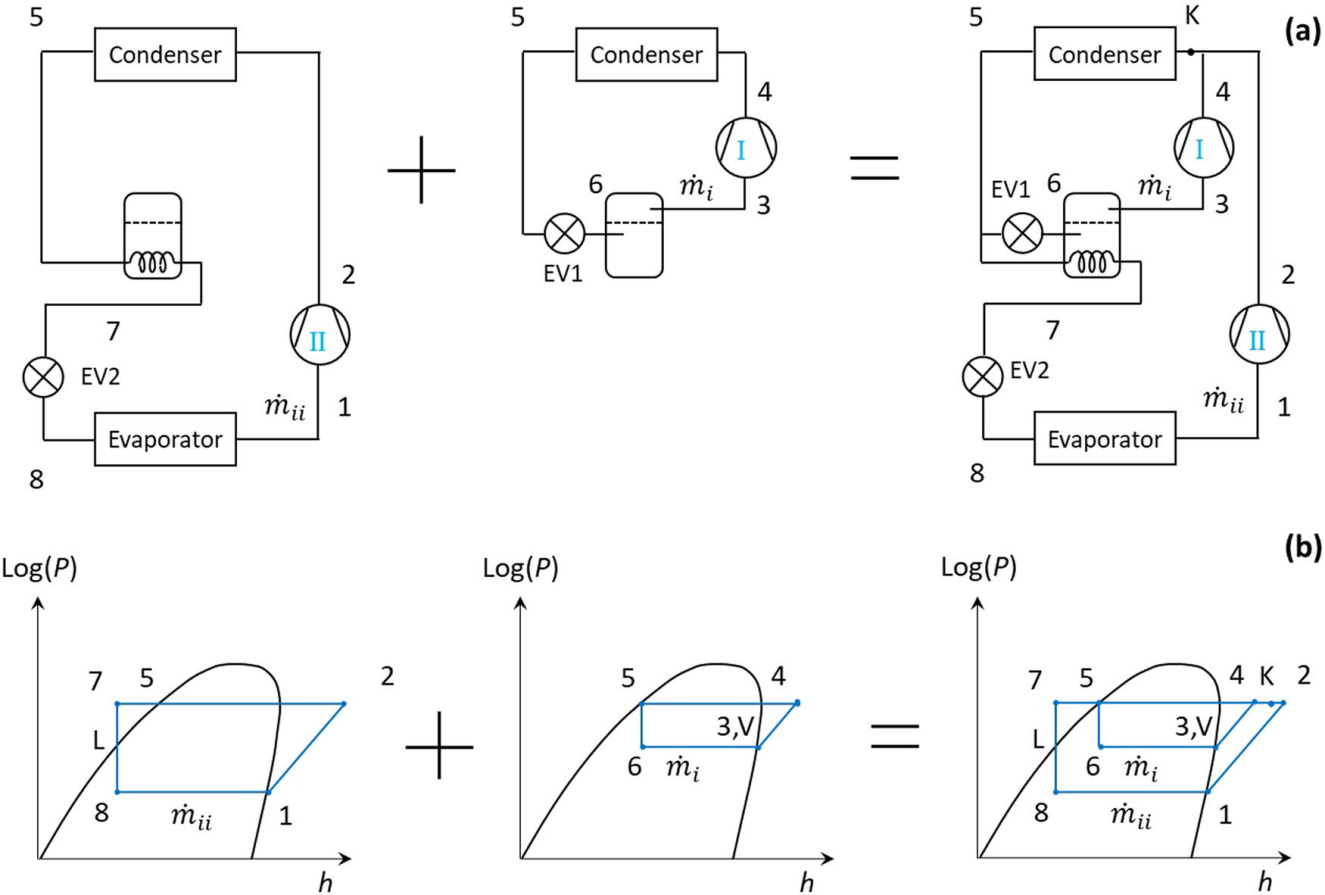
The proposed cycle superposition approach. **a** Cycle layout; (**b**) pressure-enthalpy diagram.

Take the SC cycle (see Fig. 1c) as an example, as shown in Fig. 10a, one stream of refrigerant with a mass flow rate of ${\dot{m}}_{i}$ executes a cycle 3–4-5–6-3, with the flash tank acting as an evaporator. Its compressor power ${\dot{W}}_{{comp},{steam}1}$ is ${\dot{m}}_{i}({h}_{4}-{h}_{3})$, and its heat production through the condenser ${\dot{Q}}_{c,{ts},{stream}1}$ is ${\dot{m}}_{i}({h}_{4}-{h}_{5})$. The other stream of refrigerant with a mass flow rate of ${\dot{m}}_{{ii}}$ executes a cycle 1–2-5–7-8-1, with the flash tank acting as a sub-cooler. Its compressor power ${\dot{W}}_{{comp},{stream}2}$ is ${\dot{m}}_{{ii}}({h}_{2}-{h}_{1})$, and its heat production through the condenser ${\dot{Q}}_{c,{ts},{stream}2}$ is ${\dot{m}}_{{ii}}({h}_{2}-{h}_{5})$. As a result, the COP of the cycle can be derived as shown later in Eq. (13). Figure 10b illustrates two single-stage heat pump cycles corresponding to the two refrigerant streams on the pressure-enthalpy (p-h) diagrams. The superposition of them leads to the p-h diagram of the two-stage SC cycle as shown in Fig. 1c.

It is interesting to compare Fig. 10 with Fig. 2 of ref. ^34^. Superimposing Fig. 2b and Fig. 2d of ref. ^34^ leads to the combined p-h diagram as shown in Fig. 10b of this paper. This demonstrates the thermodynamic similarity between the flexible heat pump cycle and the two-stage heat pump cycle with full subcooling. The cycle superposition approach provides a new perspective to understand multistage heat pump cycles, as well as the flexible heat pump cycle.

Following the proposed cycle superposition approach, we can quickly derive a generic model for all two-stage cycle layouts as shown in Fig. 1.

The total heat transfer rate through the condenser can be expressed as

5 $${\dot{Q}}_{c,{ts}}={\dot{Q}}_{c,{ts},{stream}1}+{\dot{Q}}_{c,{ts},{stream}2}$$

According to Fig. 1b to h, Eq. (5) can be expressed as

6 $${\dot{Q}}_{c,{ts}}=\left\{\begin{array}{c}\left({\dot{m}}_{i}+{\dot{m}}_{{ii}}\right)\left({h}_{4}-{h}_{5}\right),{{{{{\rm{ IC;SC}}}}}}+{{{{{\rm{PIC;FGR}}}}}}+{{{{{\rm{PIC;SC}}}}}}+{{{{{\rm{FIC;FGR}}}}}}+{{{{{\rm{FIC}}}}}}\\ {\dot{m}}_{i}\left({h}_{4}-{h}_{5}\right)+{\dot{m}}_{{ii}}\left({h}_{2}-{h}_{5}\right),{{{{{\rm{SC;FGR}}}}}}\hfill\end{array}\right..$$

Here, subscript c and ts represent condenser and two-stage, respectively. Subscripts 1 to 8, L, V, and K represent the state points along the two-stage cycles.

For SC and FGR cycle layouts, two refrigerant streams mix to reach state K before entering the condenser, and the mixing processes can be expressed as

7 $${\dot{m}}_{i}{h}_{4}+{\dot{m}}_{{ii}}{h}_{2}=\left({\dot{m}}_{i}+{\dot{m}}_{{ii}}\right){h}_{K}$$

Substituting Eq. (7) into Eq. (6) leads to

8 $${\dot{Q}}_{c,{ts}}=\left\{\begin{array}{c}\left({\dot{m}}_{i}+{\dot{m}}_{{ii}}\right)\left({h}_{4}-{h}_{5}\right),{{{{{\rm{ IC;SC}}}}}}+{{{{{\rm{PIC;FGR}}}}}}+{{{{{\rm{PIC;SC}}}}}}+{{{{{\rm{FIC;FGR}}}}}}+{{{{{\rm{FIC}}}}}}\\ \left({\dot{m}}_{i}+{\dot{m}}_{{ii}}\right)\left({h}_{K}-{h}_{5}\right),{{{{{\rm{ SC;FGR}}}}}}\hfill\end{array}\right..$$

For all two-stage cycle layouts, the heat transfer rate through the evaporator can be expressed as

9 $${\dot{Q}}_{e,{ts}}={\dot{m}}_{{ii}}({h}_{1}-{h}_{8}).$$

The power consumed by the stream of refrigerant ${\dot{m}}_{i}$ can be written as

10 $${\dot{W}}_{{comp},{steam}1}={\dot{m}}_{i}\left({h}_{4}-{h}_{3}\right).$$

The power consumed by the stream of refrigerant ${\dot{m}}_{{ii}}$ can be written as

11 $${\dot{W}}_{{comp},{stream}2}=\left\{\begin{array}{c}{\dot{m}}_{{ii}}\left({h}_{4}-{h}_{3}+{h}_{2}-{h}_{1}\right),{{{{{\rm{ IC;SC}}}}}}+{{{{{\rm{PIC;FGR}}}}}}+{{{{{\rm{PIC;SC}}}}}}+{{{{{\rm{FIC;FGR}}}}}}+{{{{{\rm{FIC}}}}}}\\ {\dot{m}}_{{ii}}\left({h}_{2}-{h}_{1}\right),{{{{{\rm{SC;FGR}}}}}}\hfill\end{array}\right.$$

The energy balance of the flash tank can be derived as

12 $$\left\{\begin{array}{c}{\dot{m}}_{i}{h}_{6}+{\dot{m}}_{{ii}}{h}_{2}=\left({\dot{m}}_{i}+{\dot{m}}_{{ii}}\right){h}_{V},{{{{{\rm{IC}}}}}}\hfill \\ \left({\dot{m}}_{i}+{\dot{m}}_{{ii}}\right){h}_{6}={\dot{m}}_{{ii}}{h}_{7}+{\dot{m}}_{i}{h}_{V},{{{{{\rm{SC;FGR;SC}}}}}}+{{{{{\rm{PIC;FGR}}}}}}+{{{{{\rm{PIC}}}}}}\hfill \\ \left({\dot{m}}_{i}+{\dot{m}}_{{ii}}\right){h}_{6}+{\dot{m}}_{{ii}}{h}_{2}={\dot{m}}_{{ii}}{h}_{7}+\left({\dot{m}}_{i}+{\dot{m}}_{{ii}}\right){h}_{V},{{{{{\rm{SC}}}}}}+{{{{{\rm{FIC;FGR}}}}}}+{{{{{\rm{FIC}}}}}}\end{array}\right..$$

For all two-stage cycle layouts as shown in Fig. 1, the heat pump COP can be derived as

13 $${{COP}}_{{ts}}=\frac{{\dot{Q}}_{c,{ts}}}{{\dot{W}}_{{comp},{stream}1}+{\dot{W}}_{{comp},{stream}2}}.$$

The COP improvement of the two-stage cycle against the standard single-stage cycle can be expressed as

14 $$\alpha =\frac{{{COP}}_{{ts}}-{{COP}}_{{ss}}}{{{COP}}_{{ss}}}\times 100 \% .$$

Substituting Eqs. (6), (10) and (11) into Eq. (13) leads to

15 $${{COP}}_{{ts}}=\left\{\begin{array}{c}\frac{\left({\dot{m}}_{i}+{\dot{m}}_{{ii}}\right)\left({h}_{4}-{h}_{5}\right)}{\left({\dot{m}}_{i}+{\dot{m}}_{{ii}}\right)\left({h}_{4}-{h}_{3}\right)+{\dot{m}}_{{ii}}\left({h}_{2}-{h}_{1}\right)},{{{{{\rm{ IC;SC}}}}}}+{{{{{\rm{PIC;FGR}}}}}}+{{{{{\rm{PIC;SC}}}}}}+{{{{{\rm{FIC;FGR}}}}}}+{{{{{\rm{FIC}}}}}}\\ \frac{\left({\dot{m}}_{i}+{\dot{m}}_{{ii}}\right)\left({h}_{K}-{h}_{5}\right)}{{\dot{m}}_{i}\left({h}_{4}-{h}_{3}\right)+{\dot{m}}_{{ii}}({h}_{2}-{h}_{1})},{{{{{\rm{ SC;FGR}}}}}}\hfill\end{array}\right..$$

The mass flow rate ratio of the two streams is defined as

16 $$\beta =\frac{{\dot{m}}_{i}}{{\dot{m}}_{{ii}}}.$$

Substituting Eq. (16) into Eq. (14) leads to

17 $${{COP}}_{{ts}}=\left\{\begin{array}{c}\frac{(1+\beta )\left({h}_{4}-{h}_{5}\right)}{(1+\beta )\left({h}_{4}-{h}_{3}\right)+\left({h}_{2}-{h}_{1}\right)},{{{{{\rm{ IC;SC}}}}}}+{{{{{\rm{PIC;FGR}}}}}}+{{{{{\rm{PIC;SC}}}}}}+{{{{{\rm{FIC;FGR}}}}}}+{{{{{\rm{FIC}}}}}}\\ \frac{(1+\beta )\left({h}_{K}-{h}_{5}\right)}{\beta \left({h}_{4}-{h}_{3}\right)+({h}_{2}-{h}_{1})},{{{{{\rm{ SC;FGR}}}}}}\hfill\end{array}\right..$$

According to Eq. (17), $\beta$ controls the systems’ COP once the refrigerant’s properties along the cycles are given. The higher the $\beta$, the higher the COP. It is therefore interesting to investigate what factors influence $\beta$.

Substituting Eq. (16) into Eq. (12) leads to

18 $$\beta = \left\{\begin{array}{cc}\frac{{{h}_{2}-h}_{V}}{{{h}_{V}-h}_{6}},\hfill & {{{{{\rm{IC}}}}}}\hfill\\ \frac{{{h}_{6}-h}_{7}}{{{h}_{V}-h}_{6}},\hfill & {{{{{\rm{SC;FGR;}}}}}}{{{{{\rm{SC}}}}}}+{{{{{\rm{PIC;FGR}}}}}}+{{{{{\rm{PIC}}}}}}\\ \frac{({{h}_{6}-h}_{7})}{{{h}_{V}-h}_{6}}+\frac{({{h}_{2}-h}_{V})}{{{h}_{V}-h}_{6}}, & {{{{{\rm{SC}}}}}}+{{{{{\rm{FIC;FGR}}}}}}+{{{{{\rm{FIC}}}}}}\hfill\end{array}\right..$$

Since ${{h}_{7}=h}_{L}$, Eq. (18) becomes

19 $$\beta = \left\{\begin{array}{cc}\frac{{{h}_{2}-h}_{V}}{{{h}_{V}-h}_{6}},\hfill & {{{{{\rm{IC}}}}}}\hfill\\ \frac{{{h}_{6}-h}_{L}}{{{h}_{V}-h}_{6}}, \hfill& {{{{{\rm{SC;FGR;}}}}}}{{{{{\rm{SC}}}}}}+{{{{{\rm{PIC;FGR}}}}}}+{{{{{\rm{PIC}}}}}}\\ \frac{({{h}_{6}-h}_{L})}{{{h}_{V}-h}_{6}}+\frac{({{h}_{2}-h}_{V})}{{{h}_{V}-h}_{6}}, & {{{{{\rm{SC}}}}}}+{{{{{\rm{FIC;FGR}}}}}}+{{{{{\rm{FIC}}}}}}\hfill\end{array}\right..$$

According to Eq. (19), there are several interesting findings.

First, once the flash tank temperature is given, ${h}_{V}$ and ${h}_{L}$ are given, the mass flow rate ratio $\beta$ becomes a function of ${h}_{2}$ and ${h}_{6}$, which are the refrigerant’s enthalpies at the outlets of Compressor II and EV1, respectively.

Second, the mass flow rate ratio $\beta$ for SC + FIC (or FGR + FIC) cycle is simply the sum of that for SC (or FGR) and IC cycles. This indicates that the combination of subcooling (or flash gas removal) and intercooling is a linearly superposition.

Third, for SC + PIC and FGR + PIC cycle layouts, mixing the saturated vapour from the flash tank with the superheated vapour from Compressor II does not affect the mass flow rate ratio $\beta$.

State 6 at the outlet of the high stage expansion valve (EV1) is in the saturated mixture region. Its position in relation to the saturated vapour and saturated liquid lines can be determined by its quality ${x}_{6}$ which is the mass fraction of vapour in the mixture, namely

20 $${x}_{6}=\frac{{m}_{V}}{{m}_{V}+{m}_{L}},$$

where $m$ is mass, subscripts V and L represent vapour and liquid, respectively.

Once the ${x}_{6}$ is given, ${h}_{6}$ can be determined as

21 $${h}_{6}={x}_{6}{h}_{V}+(1-{x}_{6}){h}_{L}.$$

Substituting Eq. (21) into Eq. (19) leads to

22 $$\beta =\left\{\begin{array}{c}\frac{1}{(1-{x}_{6})}\frac{{{h}_{2}-h}_{V}}{({h}_{V}-{h}_{L})},{{{{{\rm{ IC}}}}}}\hfill\\ \frac{{x}_{6}}{1-{x}_{6}},{{{{{\rm{ SC;FGR;}}}}}}{{{{{\rm{SC}}}}}}+{{{{{\rm{PIC;FGR}}}}}}+{{{{{\rm{PIC}}}}}}\hfill\\ \frac{{x}_{6}}{1-{x}_{6}}+\frac{1}{(1-{x}_{6})}\frac{{{h}_{2}-h}_{V}}{({h}_{V}-{h}_{L})},{{{{{\rm{ }}}}}}{{{{{\rm{SC}}}}}}+{{{{{\rm{FIC;FGR}}}}}}+{{{{{\rm{FIC}}}}}}\end{array}\right.$$

According to Fig. 1, ${h}_{2}$ is the enthalpy at the outlet of Compressor II, hence $({{h}_{2}-h}_{V})$ represents the degree of superheat at the outlet of Compressor II.

### Refrigerant type: dry, isentropic, and wet refrigerants

It is therefore interesting to investigate how the characteristics of refrigerants will affect $\beta$, and consequently the cycles’ ${{COP}}_{{ts}}$. Figure 9 presents the saturation domes of three typical refrigerants on temperature-entropy diagrams. As shown in Fig. 9a, for some refrigerants such as ammonia, the slope of the saturated vapour line is negative. An isentropic compression starting from a saturation vapour state always ends in a superheated state, and they are denoted as dry refrigerants in this paper. To the contrary, for another group of refrigerants such as R2272a, the slope of their saturated vapour line is positive, as shown in Fig. 9c. An isentropic compression starting from a saturation vapour state always ends as a saturated mixture, and they are denoted as wet refrigerants. For a third group of refrigerants such as R124, the slope of their saturated vapour line is almost infinite, as shown in Fig. 9b. As a result, their saturated vapour lines are in parallel with isentropic lines, and they are called as isentropic refrigerants.

It should be noted that, in this paper, the dry, wet and isentropic refrigerants are defined according to the end condition of an isentropic compression process starting from a saturation vapour state for heat pumps or refrigerators. It is different from the definition of dry, wet and isentropic refrigerants by the organic Rankine cycle research community, which are defined according to the exit condition of the isentropic expansion process^36^.

According to Eq. (22), for layouts SC, FGR, SC + PIC, and FGR + PIC, $\beta$ is just a function of ${x}_{6}$. The larger the value of ${x}_{6},$ the larger the $\beta$, and consequently the larger the ${{COP}}_{{ts}}$ according to Eq. (17). However, ${x}_{6}$ strongly depends on the shape of the saturation dome of the refrigerants. According to Fig. 9, ${x}_{6}$ is relatively small for dry refrigerants, and it is modest for isentropic refrigerants, but is large for wet refrigerants. Therefore, it can be inferred that these four cycles can achieve the highest COP improvement for wet refrigerants, a modest improvement for isentropic refrigerants, and the least improvement for dry refrigerants.

However, the situation is more complicated for the cycle layout IC as shown in Fig. 1b because the degree of superheat $({h}_{2}-{h}_{v})$ can be positive, zero, or even negative, depending on the slope of the saturated vapour line.

For dry refrigerants, an isentropic compression process starting from a saturated vapour state will always end in a superheated region. Hence, $({h}_{2}-{h}_{v})\, > \, 0$, as a result $\beta \, > \, 0$. In this case, intercooling may potentially improve COP according to Eq. (19).

For isentropic refrigerants, the saturation vapour line is in parallel to the isentropic lines. An isentropic compression from a saturated vapour state will coincide with the saturated vapour line, so the refrigerant remains as saturated vapour during the compression, hence${h}_{2}-{h}_{v}=0$. As a result, $\beta =0$, consequently ${\dot{m}}_{i}=0$. Since there is no superheat at the exit of the compressor, intercooling does not increase or decrease the cycle’s COP.

For wet fluids, an isentropic compression process starting from a saturated vapour state will end in the saturated mixture region, as a result $({h}_{2}-{h}_{v}) \, < \, 0$ and $\beta \, < \, 0$. Mathematically, refrigerant flash intercooling could lead to a negative benefit. In practice, a few degrees of superheat are required at the inlet of the compressor to avoid condensation in the compressors during compression. Therefore, intercooling should not be used.

Finally, according to Eqs. (6)–(22), for each selected refrigerant, once the evaporator, condenser and flash tank conditions are defined, SC and FGR cycles will have the same set of equations and state points. Therefore, the FGR cycle is thermodynamically similar to the SC cycle with full subcooling. Similarly, it can be seen that layout FGR + PIC is similar to SC + PIC, and that FGR + FIC is similar to SC + FIC.

### Heat production contribution ratio

Since the heat production of the condenser is the desired output of a heat pump, it is therefore interesting to investigate the ratio of heat production contributed by the two refrigerant streams, and it can be defined as

23 $$\varepsilon =\frac{{Q}_{c,{ts},{stream}1}}{{Q}_{c,{ts},{stream}2}},$$

where ${Q}_{c,{ts},{stream}1}$ and ${Q}_{c,{ts},{stream}2}$ are the heat production of the condenser contributed by refrigerant mass flow rates ${\dot{m}}_{i}$ and ${\dot{m}}_{{ii}}$, respectively.

For two-stage cycles as shown in Fig. 1, the two streams of refrigerants, ${\dot{m}}_{i}$ and ${\dot{m}}_{{ii}}$, co-exist. According to Eq. (5), dividing the numerator and denominator of the right-hand side of Eq. (23) by operational time leads to

24 $$\varepsilon =\frac{{\dot{Q}}_{c,{ts},{stream}1}}{{\dot{Q}}_{c,{ts},{stream}2}}.$$

According to Fig. 1, in addition to the flash tank, some heat has also been transferred from refrigerant stream ${\dot{m}}_{{ii}}$ to stream ${\dot{m}}_{i}$ during the mixing process, either before entering the high-stage compressor (as shown in Figs. 1b, e–h) or before entering the condenser (as shown in Figs. 1c, d). Considering this extra heat transfer, Eq. (24) can be expressed as,

25 $$\varepsilon =\left\{\begin{array}{c}\frac{{\dot{m}}_{i}\left[\left({h}_{4}-{h}_{5}\right)-\left({h}_{3}-{h}_{v}\right)\right]}{{\dot{m}}_{{ii}}\left[\left({h}_{4}-{h}_{5}\right)+\left({h}_{2}-{h}_{3}\right)\right]},{{{{{\rm{IC;SC}}}}}}+{{{{{\rm{PIC;FGR}}}}}}+{{{{{\rm{PIC;SC}}}}}}+{{{{{\rm{FIC;FGR}}}}}}+{{{{{\rm{FIC}}}}}}\\ \frac{{\dot{m}}_{i}\left({h}_{4}-{h}_{5}\right)}{{\dot{m}}_{{ii}}\left({h}_{2}-{h}_{5}\right)},{{{{{\rm{ SC;FGR}}}}}}\hfill\end{array}\right..$$

Substituting Eq. (16) into Eq. (25) leads to

26 $$\varepsilon =\left\{\begin{array}{c}\frac{\left[\left({h}_{4}-{h}_{5}\right)-\left({h}_{3}-{h}_{v}\right)\right]}{\left[\left({h}_{4}-{h}_{5}\right)+\left({h}_{2}-{h}_{3}\right)\right]}\beta ,{{{{{\rm{ IC;SC}}}}}}+{{{{{\rm{PIC;FGR}}}}}}+{{{{{\rm{PIC;SC}}}}}}+{{{{{\rm{FIC;FGR}}}}}}+{{{{{\rm{FIC}}}}}}\\ \frac{\left({h}_{4}-{h}_{5}\right)}{\left({h}_{2}-{h}_{5}\right)}\beta ,{{{{{\rm{ SC;FGR}}}}}}\hfill \end{array}\right..$$

For isentropic refrigerants,

27 $$\left\{\begin{array}{c}{h}_{2}={h}_{3}={h}_{v}, {{{{{\rm{IC;SC}}}}}}+{{{{{\rm{PIC;FGR}}}}}}+{{{{{\rm{PIC;SC}}}}}}+{{{{{\rm{FIC;FGR}}}}}}+{{{{{\rm{FIC}}}}}}\\ {h}_{2}={h}_{4}, {{{{{\rm{SC;FGR}}}}}}\hfill\end{array}\right..$$

Substituting Eq. (27) into Eq. (26) leads to

28 $$\varepsilon =\beta .$$

For dry refrigerants,

29 $$\left\{\begin{array}{c}{h}_{2} \, > \, {h}_{3} \, > \, {h}_{v}, {{{{{\rm{IC;SC}}}}}}+{{{{{\rm{PIC;FGR}}}}}}+{{{{{\rm{PIC;SC}}}}}}+{{{{{\rm{FIC;FGR}}}}}}+{{{{{\rm{FIC}}}}}}\\ {h}_{2} \, > \, {h}_{4}, {{{{{\rm{SC;FGR}}}}}}\hfill\end{array}\right..$$

Substituting Eq. (29) into Eq. (26) leads to

30 $$\varepsilon \, < \, \beta .$$

For wet refrigerants, an isentropic compression from a saturated vapour ends in the two-phase region, which is not practical, but mathematically we can still have

31 $$\left\{\begin{array}{c}{h}_{2} < {h}_{3} < {h}_{v}, {{{{{\rm{IC;SC}}}}}}+{{{{{\rm{PIC;FGR}}}}}}+{{{{{\rm{PIC;SC}}}}}}+{{{{{\rm{FIC;FGR}}}}}}+{{{{{\rm{FIC}}}}}}\\ {h}_{2} < {h}_{4}, {{{{{\rm{SC;FGR}}}}}}\hfill\end{array}\right..$$

Substituting Eq. (31) into Eq. (26) leads to

32 $$\varepsilon \, > \, \beta .$$

Nevertheless, when mixing the two streams of refrigerant vapour, the resultant heat transfer from the refrigerant stream ${\dot{m}}_{{ii}}$ to ${\dot{m}}_{i}$ is always much less than the heat transfer via the flash tank. Consequently, irrespective of refrigerant type, Eq. (26) can be approximated as

33 $$\varepsilon \cong \beta .$$

Equation (33) shows that, for these two-stage heat pump cycles, the ratio of refrigerant mass flow rates $\beta$ approximates the ratio of heat production contributed by the two component cycles.

It is worth mentioning that, for the flexible heat pump cycle, the ratio of operation time between the charging and discharging modes was revealed as a key parameter controlling the COP improvement, which is defined as^34^

34 $${\beta }_{{flexi}}=\frac{\Delta {t}_{{discharge}}}{\Delta {t}_{{charge}}}.$$

For the flexible heat pump cycle, the total heat production by the condenser is defined as^34^

35 $${Q}_{c,{flexi}}={{Q}_{c,{flexi},{charge}}+Q}_{c,{flexi},{discharge}},$$

where ${Q}_{c,{flexi},{discharge}}$ and ${Q}_{c,{flexi},{charge}}$ are the condenser’s heat production during discharging mode and charging mode, respectively.

The heat production rate of the condenser^34^, ${\dot{Q}}_{c,{flexi}}$, is the same for both charging and discharging modes. Hence, ${Q}_{c,{flexi},{discharge}}$ and ${Q}_{c,{flexi},{charge}}$ can be expressed as Eqs. (36) and (37), respectively^34^.

36 $${Q}_{c,{flexi},{discharge}}={\dot{Q}}_{c,{flexi}}\Delta {t}_{{discharge}}$$

37 $${Q}_{c,{flexi},{charge}}={\dot{Q}}_{c,{flexi}}\Delta {t}_{{charge}}$$

It should be noted that the flexible heat pump operates on the charging mode and discharging mode alternatively^34^. Like Eq. (23), for a flexible heat pump, the condenser’s heat production ratio of the discharging mode and charging mode can be defined as

38 $${\varepsilon }_{{flexi}}=\frac{{Q}_{c,{flexi},{discharge}}}{{Q}_{c,{flexi},{charge}}},$$

Substituting Eqs. (36) and (37) into Eq. (38)

39 $${\varepsilon }_{{flexi}}={\beta }_{{flexi}}.$$

Therefore, ${\beta }_{{flexi}}$ essentially is the ratio of heat produced by the two operational modes of the flexible heat pump cycle.

According to Eq. (23) and Eq. (38), $\varepsilon$ of the two-stages cycles and ${\varepsilon }_{{flexi}}$ of the flexible heat pump cycle are fundamentally similar, representing the ratio of heat production between the high COP component cycle (or mode) and the low COP component cycle (or mode). Furthermore, there is a strong similarity between Eqs. (33) and (39), suggesting an underlying similarity between two-stage heat pump cycles and the flexible heat pump cycle.

## Data Availability

The datasets generated and/or analysed during the current study are available within the paper. Other materials and data are available from the corresponding author, Z.Y., upon reasonable request.
